# Comparing ATP values with bacterial contamination in a nursing home

**DOI:** 10.1186/2047-2994-4-S1-P28

**Published:** 2015-06-16

**Authors:** IT Overdevest, I Willemsen, YJ Hendriks, CJ Verhulst, JA Kluytmans

**Affiliations:** 1 Laboratory for Medical Microbiology, Stichting PAMM, Veldhoven, Netherlands; 2 Laboratory for Microbiology and Infection Control, Amphia hospital, Breda, Netherlands; 3 Laboratory for Medical Microbiology and Immunology, St. Elisabeth Hospital, Tilburg, Netherlands; 4 Thebe Long term care facilities, Breda, Netherlands; 5 Julius Center for Health Science and Primary Care, University medical Center, Utrecht, Netherlands

## Introduction

Bacterial surface contamination is an important reservoir for micro-organisms and a potential route for transmission. ATP bioluminescence is a relatively new method to assess environmental contamination, and gives direct results.

## Objectives

We compared ATP bioluminescence with aerobic colony count and presence of ESBL-producing *Enterobacteriaceae*.

## Methods

The study was performed in a nursing home in the Netherlands during an outbreak of ESBL-producing *Enterobacteriaceae*, mainly Escherichia coli. During 5 consecutive surveys, ATP measurements (3M, Zoeterwoude, The Netherlands) were performed on several predefined surfaces. ATP measurements were combined with aerobic colony count, using Rodac agar plates in 2 surveys, and selective culture for ESBL-producing Enterobacteriaceae in 3 surveys. ATP bioluminescence was expressed in relative light units (RLU), AAC was expressed in cfu/cm^2^;for the analysis a Natural Logarithmic transformation was performed to obtain an normal distribution of the values (LnRLU and LnCFU).

## Results

In total we performed 483 ATP-measurements, 197 ACC cultures and 285 ESBL-selective cultures. Toilet seats were significantly associated with higher RLU (*P*<0.001), higher prevalence of more than 40 cfu/cm^2^on aerobic colony count, but not with difference in prevalence of ESBL-producing *Enterobacteriaceae* (*P*=0.31), compared to water taps, kitchen- and living room areas.

The ATP and ACC results of the 98 paired samples showed a good correlation between LnRLU and LnCFU(R^2^=0.277; *P*<0.001). Furthermore, LnRLU of the sample sites which yielded an ESBL-producing *Enterobacteriaceae* was significantly higher than of other the sites (*P*=0.016).

## Conclusion

Our study showed that ATP bioluminescence has a good correlation with bacterial contamination determined of surfaces. Furthermore, we did find a significant correlation between LnRLU and surface contamination with ESBL-producing *Enterobacteriaceae*. In conclusion, ATP bioluminescence is a valid replacement for measuring the bacterial contamination of surfaces, and can be a useful tool for direct feedback to assess cleaning properties.

## Disclosure of interest

I. Overdevest: None declared, I. Willemsen: None declared, Y. Hendriks: None declared, C. Verhulst: None declared, J. Kluytmans Consultant for: Pfizer, biomerieux, 3M.

